# Breastfeeding-Related Practices in Rural Ethiopia: Colostrum Avoidance

**DOI:** 10.3390/nu15092177

**Published:** 2023-05-03

**Authors:** M. Ascensión Olcina Simón, Rosita Rotella, Jose M. Soriano, Agustin Llopis-Gonzalez, Isabel Peraita-Costa, María Morales-Suarez-Varela

**Affiliations:** 1 MOS Solidaria, Avda. Blasco Ibáñez, 5-8º Puerta 16, 46400 Cullera, Spain; 7alenar@gmail.com; 2 Unit of Preventive Medicine and Public Health, Department of Preventive Medicine and Public Health, Food Sciences, Toxicology and Forensic Medicine, University of Valencia, Avda. Vicent Andres Estelles s/n, 46100 Burjassot, Spain; rotella@alumni.uv.es (R.R.); agustin.llopis@uv.es (A.L.-G.); isabel.peraita@uv.es (I.P.-C.); 3 Observatory of Nutrition and Food Safety for Developing Countries, Food & Health Lab, Institute of Materials Science, University of Valencia, Carrer Catedrático Agustín Escardino 9, 46980 Paterna, Spain; jose.soriano@uv.es; 4 Joint Research Unit on Endocrinology, Nutrition and Clinical Dietetics, University of Valencia-Health Research Institute La Fe, Avda. Fernando Abril Martorell, 106, 46026 Valencia, Spain; 5 CIBER in Epidemiology and Public Health (CIBERESP), Institute of Health Carlos III, Avda. Monforte de Lemos 3-5, Pabellón 11, Planta 0, 28029 Madrid, Spain

**Keywords:** colostrum, breastfeeding, prelacteal feeding, Ethiopia

## Abstract

The practices of colostrum avoidance and prelacteal feeding, which are common in many developing countries, including Ethiopia, are firmly rooted in ancient traditions. The main objective of this work is to identify the prevalence of colostrum avoidance and study its associated factors among mothers of children aged less than 2 years old in the Oromia region of Ethiopia. A cross-sectional study on the practice of colostrum avoidance/prelacteal feeding was conducted in a rural community with 114 mothers of children under 2 years old. Our results reflected that colostrum avoidance and prelacteal feeding were practiced by 56.1% of mothers. The percentage of women who started breastfeeding in the first hour after birth, as recommended by the WHO, was 2.6%. Of the women who practiced colostrum avoidance, 67.2% gave birth at home, and 65.6% were attended by relatives. The likelihood of avoiding colostrum increases in mothers who have a lower educational level, who did not receive health care at the time of delivery, who think that colostrum is dirty and dangerous and who did not receive information about breastfeeding from healthcare professionals. The knowledge emanating from this work may be useful in designing new breastfeeding education programs and/or interventions in Ethiopia and other developing countries.

## 1. Introduction

Breastfeeding is an essential practice of optimal nutrition in the early life of a child and one of the most important factors for child survival and the prevention of childhood infections [1]. The World Health Organization (WHO) recommends that infants be exclusively breastfed for the first six months of life [2]. Exclusive breastfeeding is defined as giving no food or drink, not even water, except breastmilk [2]. The optimal breastfeeding practice includes initiation within the first hour after birth and continued breastfeeding for up to two years [3,4]. Colostrum is the first secretion produced by the mammary gland after childbirth, is available to the neonate in the first two to three days following birth [2] and is sometimes referred to as “golden milk” [5] due to its nutritional properties as a complete form of nutrition for newborns. It has been shown to be a protective factor against childhood malnutrition [6] and to deliver natural immunity against many bacteria and viruses by establishing microbiota in the newborn’s gut [7,8]. Colostrum avoidance, which is defined as discarding colostrum within the first three days postpartum and entails the delayed initiation of breastfeeding, pumping and discarding colostrum, and/or wet nursing [9], deprives the neonate of nutrients and immunoglobulins, causing a reduction in the priming of the gastrointestinal tract and increasing the risk of infant morbidity and mortality [10]. Colostrum avoidance is a common practice in many developing countries of the world [11], including Ethiopia [9]. In Ethiopia, colostrum and breast milk are considered two distinctly separate substances known, respectively, as ‘inger’ and ‘yetut wotet’, and women will wait until they observe the characteristics of ‘yetut wotet’ to start breastfeeding [9]. During this period between birth and the establishment of breastfeeding, during which colostrum or ‘inger’ is discarded, a practice known as prelacteal feeding takes place. Prelacteal feeding, known in Amharic as ‘makamesha’, is the practice of feeding the child any solid or liquid foods other than breast milk during the first three days after birth [12]. Some of the solid foods given to the newborns include ‘injera’, ‘shiro’, ‘genfo’ and ‘faffa’. The basis of any Ethiopian meal is a teff flour flatbread named ‘injera or enjera’. ‘Shiro’ is a dish prepared with chickpea flour, water, oil, onions and a spice called ‘berbere’, which is very commonly used in Ethiopia. ‘Genfo’ is the Amharic name given to a breakfast porridge, and ‘faffa’ is a mix of fortified corn and soya bean flours used to supplement the diet of children suffering from malnutrition. It is estimated that 90% or more of children under ten years old are multidimensionally poor in Ethiopia [13]. Rural areas are particularly disadvantaged due primarily to their lack of access to safe drinking water, sanitation and/or electricity. Most of the inhabitants of these areas suffer from poor nutrition, repeated infection and inadequate psychosocial stimulation given to the lack of access to education making it particularly difficult to achieve an improvement of the situation [14]. The aim of this study was to identify the prevalence of the breastfeeding-related practice of colostrum avoidance and study its associated factors among mothers of children aged less than two years old in two rural villages of the Oromia region of Ethiopia. 

## 2. Materials and Methods

### 2.1. Research Design

This cross-sectional community-based study carried out in two rural Ethiopian villages was approved by the Ethics Committee of Research in Humans of the Ethics Commission in Experimental Research of the University of Valencia (Register code: 1256147). It is in line with the ethical principles established by the Declaration of Helsinki [15], the U.S. National Bioethics Advisory Commission [16] and the European Commission [17]. It also follows the International Compilation of Human Research Standards applied to Ethiopia based on Proclamation 60/1999 (Section 21) and National Health Research Ethics Review Guidelines [18]. The design of the study is in line with its objective of determining the prevalence of colostrum avoidance and detecting associated factors. This work has been prepared in accordance with the STROBE guidelines for observational studies [19]. All the women participating in this study were recruited under the same inclusion and exclusion criteria. They were all interviewed in their homes following the same previously established protocol. The same interview questionnaire was used, which allows us to calculate the prevalence of the different variables studied. Subsequently, participants were stratified by WHO recommendations on the initiation of breastfeeding and the practice of prelacteal feeding. Those women who rejected colostrum in their lactation and practiced prelacteal feeding were considered the avoidance group, and those who did not reject colostrum during lactation and did not practice prelacteal feeding were considered the non-avoidance group. The outcome variable was colostrum avoidance during the early breastfeeding period.

### 2.2. Setting and Relevant Context

This study was carried out, in collaboration with The Missionary Community of Saint Paul the Apostle (MCSPA) [20], and the Spanish NGO MOS SOLIDARIA (MOSS) [21], in the villages of Andode and Muke Turi where MCSPA operates. Andode is found in the Anger Guten Valley in the Gida-Kiremu district of the Oromia region, 331 km from Addis Ababa. Muke Turi is in the North Shoa region, 78 km northeast of Addis Ababa, and populated mainly by the Oromo ethnic people. The MCSPA has implemented a comprehensive program for development in the area comprising 3 health posts (Angar, Andode and Fite Bako) that care for an estimated 12,000 people and 3 nurseries (Guten, Gida and Andode) for around 450 children. It also runs the “Saint Joseph Mother and Child Center” in Muke Turi and a Nutritional Unit in Andode, where around 450 children 4 to 6 years old receive 2 nutritious meals per day, medical care and reading and writing lessons.

### 2.3. Participants

A sample size calculation was performed using the following values: 95% confidence level, 5% margin of error, 10% population proportion and 500 for population size. The value for the population proportion was chosen taking into consideration the prevalence of colostrum avoidance reported in previous studies in Ethiopia, and the population number is an estimation of the number of women of child-bearing age made using information collected by the MCSPA as no official demographic data for Andode and Muke Turi are available. The necessary sample was 109 women. The households that had potentially eligible study participants were identified by the MCSPA using the health extension workers’ logbook. This helped to identify the initial convenience sample of woman that could meet the inclusion criteria of having a live child under 2 years of age and the exclusion criteria of not having given informed consent, providing unreliable responses and/or missing 30% of the responses. Once all potentially eligible women were identified from the population records kept by the MCSPA, a systematic random sampling technique (women that attended the MCSPA posts for any reason on days the project collaborators were present in each village) was used to choose which women would be invited to participate. The total number of potential participants (women of child-bearing age) was around 500; the actual number of women with a live child under 2 years of age is unknown as, as stated previously, no official demographic data are available for the studied populations. A total of 114 women were invited to participate in the study. All women invited to participate in the study accepted the offer, and, therefore, 114 mothers who had children aged less than 2 years old were included in the study. Participation was around 23%, which is about what was expected, taking into consideration previous experiences with this population.

### 2.4. Data Collection

Data were collected in situ in November 2020 by project collaborators from the University of Valencia with the aid of MCSPA, MOSS and local collaborators that served mainly as translators. Collection took place after the women were attended by the MCSPA and MOSS in a room ceded by the MCSPA within their installations in both villages for this specific purpose. A semi-structured questionnaire, which was not pre-tested, was used for data collection. It was created specifically for this study after reviewing the experiences of the local collaborators, the currently available literature on the topic and the possible association of breastfeeding practices with infant malnutrition. This questionnaire was administered during an individual face-to-face 30 min interview with the mothers. All possible participants were informed about the objectives of the study and the data confidentiality standards and had informed consent documents verbally translated by a native Ethiopian translator fluent in English and Amharic. For the women unable to write, an ink-stained fingerprint was used to indicate their agreement to the informed consent document in place of a signature. An identification number was given to every participant and collected data were anonymized in order to ensure confidentiality. Completed questionnaires were kept at all times by the collaborators from the University of Valencia in secure conditions (locked box) and are now stored under lock and key at the University of Valencia.

### 2.5. Measurement

The aim of the questionnaire was to collect data on if the mothers received antenatal care and/or infant nutritional guidance during pregnancy and to determine their infant feeding practices in the first three days postpartum. The data collection questionnaire used comprised three main sections. The first section included general personal and sociodemographic characteristics of the mothers such as their age, level of education, number of live children and delivery problems. The second section collected data specifically related to infant feeding practices, such as supplementary feeding, to understand when the child started to receive foods different from human milk and what kind of foods they received. The absence of a free-access hospital or conventional health care center in the area impeded the collection of any sort of official medical history. The third section of the questionnaire focused on the living conditions of the women such as building materials utilized for the construction of the home, sanitary conditions and the availability of drinking water. The overcrowding rate was calculated by dividing the square meters of the home by the number of people living in it. In this study, the outcome variable was the practice of colostrum avoidance among mothers of children aged less than two years old. The independent variables included the mother’s characteristics, household characteristics and child’s sex. Mothers were divided into two groups depending on their colostrum avoidance status. Women who avoided feeding colostrum to their infant formed the avoidance group, and the non-avoidance group was composed of the women who fed colostrum to their infant.

### 2.6. Data Analysis

After the quantitative data on the printed questionnaire form were completed and checked for consistency, the data were scrubbed, coded and entered into IBM SPSS Statistics (Version 26). Qualitative data were transcribed into English text. The data analysis, according to the project objective, was performed using a key question from the interview regarding colostrum avoidance at the initiation of breastfeeding. Frequencies and percentages were used to describe the prevalence of colostrum avoidance. Categorical variables were described with frequency and percentages, and the comparison among the groups, according to colostrum avoidance, was performed using Pearson’s chi-square test with Yates’s correction. Fisher’s exact test was used when the expected count was less than 5. Continuous variables were described as means and standard deviation (SDs), after which normality was evaluated with the Kolmogorov–Smirnov test. An independent sample *t*-test was used to compare the groups. Crude odds ratios (ORs) were reported with 95% confidence interval (95% CI). Variables at *p*-value < 0.05 in the analysis were concluded as factors associated with colostrum avoidance.

## 3. Results

### 3.1. Characteristics of the Sample

The prevalence of colostrum avoidance was 56.1% (*n* = 64). The approximate age of the participants ranged from 18 to 45 years with a mean of 25.3 ± 5.0 years old. The mean in the avoidance group was 27.3 ± 6.6 years old, and the mean in the non-avoidance group was 27.4 ± 6.4 years old, with no significant differences (*p* = 0.870) between the groups. A general description of the participants is shown in Table 1. 

Overall, 78.1% of the participants interviewed were illiterate and 7.0% had a secondary education. Among illiterate participants, 57.3% avoided colostrum, while the avoidance rate in literate participants was 52.0%. While the COR is higher for the illiterate participants than for those able to read and write, the sample is small and lacks the power to make any definitive conclusions regarding education. In addition, if the sample is collapsed into literate and illiterate groups, then the result does not differ based on education level. Significant differences were observed among the groups (*p* = 0.001) for parity. Most of the participants in the study were multiparous, and women who had more than one child more frequently avoided colostrum than participants who had one child, which was confirmed with a crude odds ratio (COR) > 1. The mean number of children was 3.5 ± 0.7 (3.9 ± 2.0 in the avoidance group and 4.0 ± 1.8 in the non-avoidance group). Overall, 84.2% of the participants were still breastfeeding their children, while 15.8% of the participants interviewed had stopped breastfeeding. No significant differences were observed among the groups. The only participants to follow WHO recommendations on the initiation of breastfeeding were in the avoidance group.

### 3.2. Living Conditions

Table 2 presents the living conditions of the participants and their families who took part in the study. The quality of the flooring material was significantly different between the groups with most participants in the avoidance group stating that the flooring consisted of soil, while the value was lower but still constituted the majority for the non-avoidance group. Additionally, significant differences between the groups regarding the number of animals in the home were not found. 

In both villages, more than 90% of the participants in the study were living in the traditional Ethiopian thatched-roof hut typical of the rural areas called ‘tukul’, in which any type of available wood, commonly eucalyptus planks, is used for wall construction and for the conical-shaped roof support and the floor of the house is plain earth. The mean number of people living in the house was 5.0 ± 1.9 (4.9 ± 0.9 in the avoidance group and 5.1 ± 1.8 in the non-avoidance group (*p* = 0.527)), while the square footage of the house was 15.5 ± 5.4 m^2^ (16.6 ± 6.0 m^2^ in the avoidance group and 14.0 ± 4.4 m^2^ in the non-avoidance group (*p* = 0.158)). From the data collected, we estimated an average of 3.4 ± 1.4 m^2^ (3.9 ± 1.6 m^2^ in the avoidance group and 2.9 ± 0.8 m^2^ in the non-avoidance group (*p* = 0.001)) of floor area per person, while the WHO literature suggests 9–10 m^2^ of floor area per person. No differences were found in regard to toilet facility, the type of animal inside the house or main source of drinking water.

### 3.3. Antenatal Care

The information collected on the health care received during pregnancy and delivery is shown in Table 3. 

More than half of all the participants received antenatal care during pregnancy. The percentage of participants who did not receive antenatal was higher in the avoidance group than in the non-avoidance group without a significant difference. Significant differences were observed for the mode of delivery and the delivery attendants. The participants who practice colostrum avoidance were more likely to have caesarean births and be attended by relatives. Around 7/10 of the participants who practice colostrum avoidance gave birth at home, while the percentage of home deliveries decreased, but not significantly, in the non-avoidance group. No differences were observed in diet supplementation.

### 3.4. Infant Feeding Practices and Beliefs

The main findings of the study regarding the participants’ knowledge and attitudes about infant feeding practices are shown in Table 4 and Table 5. 

Significant differences were observed among the groups in regard to breastfeeding beliefs but not for infant feeding practices. The percentage of participants who did not discard colostrum that said that colostrum stimulates milk production or that it is important for the infant was less than 30%, while less than 5% of those that did discard colostrum agreed with this statement. Among those that discarded colostrum, around 80% thought that colostrum was dirty and could be dangerous for the newborn. These beliefs are significantly associated with avoiding colostrum (COR = 6.65). The participants in this study declared that, during the first days after giving birth, they would wet their breast with hot water and manually massage it in order to extract the colostrum, which they would then discard. However, none of them could explain the reason for this practice; it was simply understood as traditional. The large percentage of participants who did not receive information about infant feeding during pregnancy learned how to feed their infants from popular traditions handed down from mother to daughter, and this characteristic makes these women have a significantly (COR = 2.43) higher probability of avoiding colostrum. This probability was similarly high in the participants who directly agreed with colostrum avoidance (COR = 5.27) or stated to not have an opinion (COR = 2.13). In general, a third of participants stated that they fed their infants with only human milk during the first three days after birth; within them, a quarter of the avoidance group had received information about breastfeeding, while in the non-avoidance group, the percentage was almost double. Of the 114 participants who answered and had children aged more than 6 months, almost all said that human milk was the main food that the infants received during the first 6 months of life. The results show that the majority of the participants started supplementary feeding at six months. The most commonly added foods to a child’s diet were ‘injera’, ‘shiro’ and ‘genfo’.

## 4. Discussion

This study revealed that the prevalence of colostrum avoidance was higher than that described by one study conducted in an urban area of Ethiopia, which reported an avoidance of 6.3% [22], and that described by studies performed in other different areas of Ethiopia, including Raya Kobo (13.5%) [23], Amibara (36.9%) [24], Goba Woreda (35.0%) [25], and rural northern Ethiopia (63%) [12]. Due to inaccessible health care, many of the mothers in our study did not receive adequate prenatal care and gave birth at home. In Andode, there is no hospital, only a health post without a pharmacy. The nearest hospital and pharmacy are 70 km away. While there is a small hospital in Muke Turi, it does not have the capability to treat any serious medical situation, and those requiring more specialized care must travel to Addis Ababa 80 km away. Additionally, health care is costly, and the majority of the women do not have insurance and are unable to cover the associated cost. In an emergency or a life or death case, the MCSPA will cover the health care cost, but antenatal care must be paid for by the women. 

A lack of prenatal care contributes to inadequate breastfeeding education and reliance on maternally transmitted, traditional infant feeding beliefs and practices. Maternal education and antenatal care have been shown to be connected to the early initiation of breastfeeding (EIBF) and exclusive breastfeeding (EBF) rates [24,26,27,28,29,30,31,32,33,34,35,36,37,38,39,40,41]. Therefore, appropriate antenatal care that includes a maternal education component on adequate breastfeeding practices may help improve rates of EIBF and EBF. Given the structure of health care in Ethiopia and the difficulty of accessing it for some of its citizens, the use of health extension workers is recommended [42]. The rate of EBF in Ethiopia is significantly under the global recommendations [42]. There are recent scientific publications from Ethiopia regarding breastfeeding practices and their associated factors available that could be used as a basis for the design of interventions geared towards improving EIBF and EBF rates [41,43]. The advantages of EIBF for both mother and infant [44], such as lower neonatal mortality [45,46], have been clearly proven. It is difficult to establish a national rate of EIBF in Ethiopia as previous studies have shown results ranging from 40% to over 80% [24,26,27,28,29,30,31,32,33,34,35,36]. However, the results of this study in a rural area are in line with those of previous studies, which have shown lower EIBF rates in women from rural areas compared to women from urban centers [24,29,30,35,47]. 

Colostrum avoidance is a common practice in Ethiopia [12,22,23,48]; however, studies [49,50] have shown varying degrees of avoidance with different regions of the country presenting rates as high as 77% or as low as 11%. Colostrum avoidance (56.14%) in this study was higher than the estimated national Ethiopian average (39.8%) [51] and that found in more developed areas of the country [22,52]. This difference in incidence is significantly associated (COR = 9.39) with a low level of education in the same areas, where 78.1% were illiterate. The participating women stated that they would actively discard colostrum by wetting their breast with hot water and pumping in the days immediately after giving birth. When asked to explain the reasoning behind this practice, they stated they followed this practice because they believed colostrum to be dirty and dangerous for the newborn or to be insufficient for the newborn because it is too similar to water. 

A systematic review and meta-analysis calculated the pooled prevalence of prelacteal feeding in Ethiopia at 25.29% with severe heterogeneity [53]. This traditional practice delays the initiation of breastfeeding and can affect the future success of breastfeeding [12,48,54,55]. This practice is more common in rural areas than in urban areas due to the lack of education regarding infant feeding and the lack of health care centers, which leads to high rates of homebirths [12,41,53,56]. The newborn intestinal tract is more permeable and vulnerable to pathogens, which may be carried in prelacteal foods [56]. This can lead to a microbial load too high to handle for the immature immune system of the infant, a situation made worse due to colostrum avoidance. Colostrum, the perfect food for a newborn to receive after birth, is essential to compensate for the immunological immaturity of the newborn intestinal tract, is low in fat [57,58] and improves the gut microbiome of the newborn [59,60].

This study has certain limitations that must be taken into consideration. The cross-sectional design has some inherent limitations regarding the nature of the association between the different factors and colostrum avoidance. While factors associated with colostrum avoidance can be determined, the nature of the relationship of these factors and the practice of colostrum avoidance cannot be established. This is the first scientific study carried out in these particular areas regarding breastfeeding practices. The questionnaire administered during the face-to-face interviews was not pre-tested and had to be adapted in situ due to limitations regarding date availability. One of the main limitations related to data availability was the fact that most of the women did not become aware of their pregnancy until the third/fourth month, and no official medical histories were available for review. The information obtained from mothers might be subject to recall bias. The sample size is also a limitation. This limitation arises mainly due to the limited time that the research team was permitted to stay in either village, the travel time between locations and the length of the interviews, which needed simultaneous translation.

## 5. Conclusions

This study allows us to identify that mothers are not well educated about correct infant feeding practices and that colostrum avoidance is still widely practiced in this rural region at a higher rate than found in previous studies carried out in other parts of Ethiopia. A low level of education and limited health care are the main factors for colostrum avoidance. Education and quality health care are central for development at every level. Education is an essential tool for improving living conditions, reducing poverty and building a food-secure world. Adequate infant feeding information and care from health professionals during and after pregnancy is still a luxury in these rural areas that most women will not be able to access. An intervention aimed at improving access to nutritional education and health care could help reduce the prevalence of colostrum avoidance in a sustainable way that could lead to improved overall infant and community health and development.

## Figures and Tables

**Table 1:** Characteristics of mothers of children aged less than 24 months in a rural area of Ethiopia.

Variable	Colostrum Avoidance								
	Total (*n* = 114)		Avoidance ^1^ (*n* = 64)		Non-Avoidance ^2^ (*n* = 50)		*p* *	Crude Odds Ratio (95% CI)	*p* *
	*n* (%) **	95% CI	*n* (%) **	95% CI	*n* (%) **	95% CI			
**Educational level**									
Secondary school	8 (7.0%)	(3.30, 13.78)	1 (1.56%)	(0.1, 9.5)	7 (14.0%)	(6.3, 27.4)	0.121	1	-
Able to read and write	17 (14.9%)	(9.17, 23.08)	12 (18.8%)	(10.5, 30.8)	5 (10.0%)	(3.7, 22.6)		16.80 (1.62, 174.53)	0.022
Illiterate	89 (78.1%)	(69.15, 85.05)	51 (79.7%)	(67.4, 88.3)	38 (76.0%)	(62.8, 86.3)		9.39 (1.11, 79.61)	0.039
**Parity**									
Multiparous	85 (74.6%)	(65.36, 82.05)	45 (70.3%)	(57.4, 80.8)	40 (80.0%)	(65.9, 89.5)	0.001	1	
Primiparous	29 (24.5%)	(17.95, 34.61)	19 (29.7%)	(19.2, 42.6)	10 (20.0%)	(10.5, 34.1)		1.69 (0.70, 4.06)	0.336
**Breastfeeding**									
Still breastfeeding	96 (84.2%)	(75.92, 90.12)	50 (78.1%)	(65.7, 87.1)	46 (92.0%)	(79.9, 97.4)	0.292	1	-
Not breastfeeding	18 (15.8%)	(9.87, 24.07)	14 (21.9%)	(12.9, 34.3)	4 (8.0%)	(2.6, 20.1)	-	3.22 (0.99, 10.49)	0.079
**Breastfeeding initiation time**									
<1 h	3 (2.6%)	(0.68, 8.07)	3 (4.7%)	(1.2, 14.0)	0 (0.0%)	(0.00, 8.9)	-	-	-
>1 h	111 (97.4%)	(91.9, 99.3)	61 (95.3%)	(86.0, 98.8)	50 (100,0%)	(91.1, 100.0)		-	-

^1^ Women who rejected colostrum in their lactation and practiced prelacteal feeding. ^2^ Women who did not reject colostrum during lactation and did not practice prelacteal feeding. * *p* value < 0.05 considered statistically significant. *p* value calculated using ANOVA or Chi-squared test. ** % by column.

**Table 2:** Living conditions of the families who took part in the study.

Variable	Colostrum Avoidance								
	Total (*n* = 114)		Avoidance ^1^ (*n* = 64)		Non-Avoidance ^2^ (*n* = 50)		*p* *	Crude Odds Ratio (95% CI)	*p* *
	*n* (%) **	95% CI	*n* (%) **	95% CI	*n* (%) **	95% CI			
**Flooring material of the house**									
Soil	90 (81.8%)	(73.1, 88.3)	56 (88.9%)	(77.8, 95.0)	34 (72.3%)	(57.1, 83.9)	0.005	1	-
Cement	7 (6.4%)	(2.8, 13.1)	5 (7.9%)	(3.0, 18.3)	2 (4.3%)	(0.7, 15.7)		1.52 (0.28, 8.26)	0.231
Mud	13 (11.8%)	(6.7, 19.7)	2 (3.2%)	(0.6, 12.0)	11 (23.4%)	(12.8, 38.4)		0.11 (0.02, 0.53)	0.004
**Toilet facility**									
Pit latrine	85 (78.0%)	(68.8, 85.1)	48 (76.2%)	(63.5, 85.6)	37 (80.4%)	(65.6, 90.1)	0.297	1	-
No Facilities	24 (22.0%)	(14.9, 31.2)	15 (23.8%)	(14.4, 36.5)	9 (19.6%)	(9.9, 34.4)		1.28 (0.51, 3.26)	0.768
**Animals inside the house**									
Yes	33 (30%)	(21.8, 39.6)	19 (30.2%)	(19.6, 43.2)	14 (29.8%)	(17.8, 45.1)	0.002	1	-
No	77 (70%)	(60.4,78.2)	44 (69.8%)	(56.8, 80.4)	33 (70.2%)	(54.9, 82.2)		1.02 (0.45, 2.32)	0.866
**Number of people living in the house**									
<4	58 (50.9%)	(41.4, 60.3)	33 (51.6%)	(38.8, 64.1)	25 (50.0%)	(35.7, 64.3)		1	-
4–8	48 (42.1%)	(33.0, 51.7)	27 (42.2%)	(30.2, 55.2)	21 (42.0%)	(28.5, 56.7)		0.97 (0.45, 2.11)	0.610
>8	8 (7.0%)	(3.3, 13.8)	4 (6.3%)	(2.0, 16.0)	4 (8.0%)	(2.6, 20.1)		0.76 (0.17, 3.33)	0.511
**m^2^ of the house**									
<10	24 (21.1%)	(14.2, 29.9)	9 (14.1%)	(7.0, 25.5)	15 (30.0%)	(18.3, 44.8)		2.62 (1.03, 6.63)	0.041
>10	90 (78.9%)	(70.1 85.8)	55 (85.9%)	(74.5, 93.0)	35 (70.0%)	(55.2, 81.7)		1	-
**Overcrowding rate ^3^**									
<3	57 (50.0%)	(40.5, 59.5)	32 (50.0%)	(37.4, 62.6)	25 (50.0%)	(35.7, 64.3)		1.21 (0.58, 2.56)	0.401
>3	57 (50.0%)	(40.5, 59.5)	32 (50.0%)	(37.4, 62.6)	25 (50.0%)	(35.7, 64.3)		1	-
**Kind of animals**							0.534		
Chickens	19 (55.9%)	(38.1, 72.4)	11 (57.9%)	(33.9, 78.9)	8 (53.3%)	(27.4, 77,7)		1	-
Goats	2 (5.9%)	(1.0, 21.1)	1 (5.3%)	(0.3, 28.1)	1 (6.7%)	(0.3, 34.0)		0.73 (0.04, 13.45)	0.591
Cats	2 (5.9%)	(1.0, 21.1)	1 (5.3%)	(0.3, 28.1)	1 (6.7%)	(0.3, 34.0)		0.73 (0.04, 13.45)	0.591
Others	11 (32.4.0%)	(18.0, 50.6)	6 (31.6%)	(13.6, 56.5)	5 (33.3%)	(13.0, 61.3)		0.87 (0.20, 3.90)	0.838
**Main source of drinking water**									
Covered well	60 (54.5%)	(44.8, 64.0)	33 (54.1%)	(40.9, 66.7)	27 (55.1%)	(40.3, 69.1)	0.667	1	-
Open well	1 (0.9%)	(0.1, 5.7)	1 (1.6%)	(0.1, 10.0)	0 (0.0%)	(0.00, 9.1)		-	-
River	49 (44.5)	(35.2, 54.3)	27 (44.3%)	(31.8, 57.5)	22 (44.9%)	(30.9, 59.7)		1.00 (0.47, 2.14)	0.855

^1^ Women who rejected colostrum in their lactation and practiced prelacteal feeding. ^2^ Women who did not reject colostrum during lactation and did not practice prelacteal feeding. ^3^ Overcrowding rate: m^2^ of the house/number of people living in the house. * *p* value < 0.05 considered statistically significant. *p* value calculated using ANOVA or Chi-squared test. ** % by column.

**Table 3:** Health care that the women interviewed received during pregnancy and information about delivery.

Variable	Colostrum Avoidance								
	Total (*n* = 114)		Avoidance ^1^ (*n* = 64)		Non-Avoidance ^2^ (*n* = 50)		*p* *	Crude Odds Ratio (95% CI)	*p* *
	*n* (%) **	95% CI	*n* (%) **	95% CI	*n* (%) **	95% CI			
**Antenatal care**									
Yes	70 (61.4%)	(51.8, 70.2)	36 (56.3%)	(43.3, 68.4)	34 (68.0%)	(53.2, 80.1)	0.635	1	-
No	44 (38.6%)	(29.8, 48.2)	28 (43.8%)	(31.6, 56,7)	16 (32.0%)	(19.9, 46.8)		1.65 (0.76, 3.58)	0.278
**Delivery mode**									
Caesarean section	7 (6.1%)	(2.71, 12.70)	4 (6.3%)	(2.0, 16.0)	3 (6.0%)	(1.6, 17.5)	0.003	1	-
Vaginal	107 (93.9%)	(87.31, 97.28)	60 (93.8%)	(84.0, 98.0)	47 (94,0%)	(82.5, 98.4)		0.96 (0.20, 4.29)	0.735
**Delivery place**									
Government hospital	18 (15.8%)	(9.87, 24.08)	9 (14.1%)	(7.0, 25.5)	9 (18.0%)	(9.0, 31.9)	0.060	1	-
Government health center	30 (26.3%)	(18.72, 35.54)	12 (18.8%)	(10.5, 30.8)	18 (36.0%)	(23.3, 50.9)		0.67 (0.21, 2.16)	0.988
Own home	66 (57.9%)	(48.28, 66.97)	43 (67.2%)	(54.2, 78.1)	23 (46.0%)	(32.1, 60.5)		1.87 (0.65, 5.36)	0.368
**Delivery attendance**									
Health professional	48 (42.1%)	(33.03, 51.72)	21 (32.8%)	(21.9, 45.8)	27 (54.0%)	(39.4, 64.9)	0.001	1	-
Trained traditional birth attendant	6 (5.3%)	(2.16, 11.57)	0 (0.0%)	(0.00, 7.1)	6 (12.0%)	(5.0, 25.0)		-	-
Relatives	56 (49.1%)	(39.70, 58.60)	42 (65.6%)	(52.6, 76.8)	14 (28.0%)	(16.7, 42.7)		3.86 (1.68, 8.86)	0.002
Nobody	4 (3.5%)	(1.13, 9.27)	1 (1.6%)	(0.1, 9.5)	3 (6.0%)	(1.6, 17.5)		0.43 (0.04, 4.42)	0.839
**Diet supplementation during pregnancy or breastfeeding**									
Yes	7 (6.2%)	(2.74, 12.80)	3 (4.7%)	(1.2, 14.0)	4 (8.2%)	(2.6, 20.5)	0.577	1	-
No	106 (93.8%)	(87.20, 97.26)	61 (95.3%)	(86.0, 98.8)	45 (91.8%)	(79.5, 97.4)		1.81 (0.39, 8.48)	0.714

^1^ Women who rejected colostrum in their lactation and practiced prelacteal feeding. ^2^ Women who did not reject colostrum during lactation and did not practice prelacteal feeding. * *p* value < 0.05 considered statistically significant. *p* value calculated using ANOVA or Chi-squared test. ** % by column.

**Table 4:** Breastfeeding practices and beliefs.

Variable	Colostrum Avoidance								
	Total (*n* = 114)		Avoidance ^1^ (*n* = 64)		Non-Avoidance ^2^ (*n* = 50)		*p* *	Crude Odds Ratio (95% CI)	*p* *
	*n* (%) **	95% CI	*n* (%) **	95% CI	*n* (%) **	95% CI			
**What does the woman think about colostrum?**									
It is important	12 (10.5%)	(5.8, 18.0)	3 (4.7%)	(1.2, 14.0)	9 (18.0%)	(9.0, 31.9)	0.001	1	-
It stimulates milk production	5 (4.4%)	(1.6, 10.4)	0 (0.0%)	(0.0, 7.1)	5 (10,0%)	(3.7, 22.6)		-	-
It is not sufficient	7 (6.1%)	(2.7, 12.7)	4 (6.3%)	(2.0, 16.0)	3 (6.0%)	(1.6, 17.5)		4.00 (0.55,29.18)	0.364
It is dirty and dangerous	74 (64.9%)	(55.4, 73.5)	51 (79.7%)	(67.4, 88.3)	23 (46.0%)	(32.1, 60.5)		6.65 (1.65,26.88)	0.009
No idea	16 (14.0%)	(8.5, 22.1)	6 (9.4%)	(3.9, 19.9)	10 (20.0%)	(10.5, 34.1)		1.80 (0.34,9.40)	0.770
**Does the woman think colostrum should be discarded?**									
Disagree	23 (20.0%)	(13.6, 29.1)	6 (9.4%)	(3.9, 19.9)	17 (34.7%)	(22.1, 49.7)	0.001	1	-
Agree	83 (73.0%)	(64.7, 81.1)	55 (85.9%)	(74.5, 93.0)	28 (57.1%)	(42.3, 70.9)		5.57 (1.98,15.68)	0.001
No idea	7 (6.0%)	(2.7, 12.8)	3 (4.7%)	(1.2, 14.0)	4 (8.2%)	(2.7, 20.5)		2.13 (0.36,12.39)	0.706

^1^ Women who rejected colostrum in their lactation and practiced prelacteal feeding. ^2^ Women who did not reject colostrum during lactation and did not practice prelacteal feeding. * *p* value < 0.05 considered statistically significant. *p* value calculated using ANOVA or Chi-squared test. ** % by column.

**Table 5:** Infant feeding knowledge and practices.

Variable	Colostrum Avoidance								
	Total (*n* = 114)		Avoidance ^1^ (*n* = 64)		Non-Avoidance ^2^ (*n* = 50)		*p* *	Crude Odds Ratio (95% CI)	*p* *
	*n* (%) **	95% CI	*n* (%) **	95% CI	*n* (%) **	95% CI			
**Did the woman receive information about infant feeding?**									
About breastfeeding only	38 (33.3%)	(25.0, 42.9)	16 (25.0%)	(15.4, 37.7)	22 (44.0%)	(30.3, 58.7)	0.102	1	-
About suppl. feeding	3 (2.6%)	(0.7, 8.1)	1 (1.6%)	(0.1, 9.5)	2 (4.0%)	(0.7 14.9)		0.69 (0.06, 8.25)	0.755
No information	72 (63.2%)	(53.6, 71.9)	46 (71.9%)	(59.0, 82.1)	26 (52.0%)	(37.6 66.1)		2.43 (1.09, 5.43)	0.047
Information on other feeding practices	1 (0.9%)	(0.1, 5.5)	1 (1.6%)	(0.1, 9.5)	0 (0.0%)	(0.00, 9.0)		-	-
**What was the main food that the child received during the first six months after birth?**									
Breast milk	100 (87.8%)	(93.8, 99.9)	56 (98.2%)	(89.4, 99.9)	44 (100.0%)	(90.0, 100.0)	0.677	1	-
Other food	1 (0.99%)	(0.1, 6.2)	1 (1.8%)	(0.1, 10.6)	0 (0.0%)	(0.0, 10.0)		-	-
**When did/will the woman start supplementary feeding?**									
2 months	1 (0.9%)	(0.0, 5.5)	0 (0.0%)	(0.0,7.1)	1 (2.0%)	(0.1, 12.0)	0.302	0.42 (0.04, 4.82) ***	0.014
5 months	2 (1.8%)	(0.3, 6.8)	1 (1.6%)	(0.1, 9.5)	1 (2.0%)	(0.1, 12.0)			
6 months	96 (84.2%)	(75.9, 90.1)	52 (81.3%)	(69.2, 89.5)	44 (88.0%)	(75.0, 95.0)		1	-
7 months	12 (10.5%)	(5.8, 18.0)	9 (14.1%)	(7.0, 25.5)	3 (6.0%)	(1.6, 17.5)		2.33 (0.69, 7.82) ****	0.704
8 months	1 (0.9%)	(0.0, 5.5)	0 (0.0%)	(0.0,7.1)	1 (2.0%)	(1.6, 17.5)			
9 months	2 (1.8%)	(0.3, 6.8)	2 (3.1%)	(0.5, 11.8)	0 (0.0%)	(0.00, 8.9)			
**What was the first food the child received/will receive?**									
Enjera	30 (29.4%)	(21.0, 39.4)	17 (27.0%)	(17.0, 39.9)	13 (33.3%)	(19.6, 50.3)	0.299	1	-
Shiro	15 (14.7%)	(8.7, 23.4)	9 (14.3%)	(7.1, 25.9)	6 (15.4%)	(6.4, 31.2)		1.15 (0.33, 4.05)	0.915
Faffa	6 (5.9%)	(2.4, 12.9)	6 (9.5%)	(3.9, 20.3)	0 (0.0%)	(0.0, 11.2)		-	-
Whatever the mother eats	9 (8.8%)	(4.4, 16.5)	7 (11.1%)	(5.0, 22.1)	2 (5.1%)	(0.9, 18.6)		2.68 (0.47, 15.09)	0.452
Porridge	24 (23.5%)	(15.9, 33.2)	13 (20.6%)	(11.9, 33.0)	11 (28.2%)	(15.5, 45.1)		0.90 (0.31, 2.66)	0.927
Genfo	11 (10.8%)	(5.8, 18.9)	8 (12.7%)	(6.0, 24.0)	3 (7.7%)	(2.0, 22.0)		2.04 (0.45, 9.24)	0.567
Other foods	7 (6.9%)	(3.0, 14.1)	3 (4.8%)	(1.2, 14.2)	4 (10.3%)	(3.3, 25.2)		0.57 (0.11, 3.02)	0.811

^1^ Women who rejected colostrum in their lactation and practiced prelacteal feeding. ^2^ Women who did not reject colostrum during lactation and did not practice prelacteal feeding. * *p* value < 0.05 considered statistically significant. *p* value calculated using ANOVA or Chi-squared test. ** % by column. *** Crude odds ratio (95% CI) for < 6 months. **** Crude odds ratio (95% CI) for >6 months.

## Data Availability

The data are not publicly available due to ethical and privacy restrictions.
